# Plasma Reactive Dicarbonyls Are Not Independently Associated With Arterial Stiffness: The Maastricht Study

**DOI:** 10.1093/ajh/hpaf068

**Published:** 2025-05-16

**Authors:** Myrthe M van der Bruggen, Marleen M J van Greevenbroek, Bart Spronck, Coen D A Stehouwer, Tammo Delhaas, Koen D Reesink, Casper G Schalkwijk

**Affiliations:** Department of Biomedical Engineering, CARIM School for Cardiovascular Diseases, Maastricht University, Maastricht, The Netherlands; Department of Internal Medicine, CARIM School for Cardiovascular Diseases, Maastricht, University Medical Centre, Maastricht, The Netherlands; Department of Biomedical Engineering, CARIM School for Cardiovascular Diseases, Maastricht University, Maastricht, The Netherlands; Department of Biomedical Engineering, School of Engineering & Applied Sciences, Yale University, New Haven, CT, USA; Department of Internal Medicine, CARIM School for Cardiovascular Diseases, Maastricht, University Medical Centre, Maastricht, The Netherlands; Department of Biomedical Engineering, CARIM School for Cardiovascular Diseases, Maastricht University, Maastricht, The Netherlands; Department of Biomedical Engineering, CARIM School for Cardiovascular Diseases, Maastricht University, Maastricht, The Netherlands; Department of Internal Medicine, CARIM School for Cardiovascular Diseases, Maastricht, University Medical Centre, Maastricht, The Netherlands

**Keywords:** ageing, blood pressure, dicarbonyls, glycation, hypertension, pulse wave velocity, type 2 diabetes mellitus, vascular stiffness

## Abstract

**BACKGROUND:**

Arterial stiffness is a strong predictor of cardiovascular diseases and all-cause mortality. Increased concentrations of highly reactive dicarbonyl compounds—methylglyoxal (MGO), glyoxal (GO), and/or 3-deoxyglucosone (3-DG)—may cause arterial stiffening via formation of advanced glycation end products, triggering maladaptive responses in vascular tissue, e.g., elastin degradation and collagen cross-linking. Therefore, we investigated in the population-based Maastricht study whether plasma MGO, GO, and 3-DG concentrations were cross-sectionally associated with carotid-to-femoral pulse wave velocity (cfPWV) and local carotid stiffness measures: pulse wave velocity (cPWV), and Young’s elastic modulus (cYEM) using standardized main variables.

**METHODS:**

Fasting dicarbonyl concentrations were determined by ultra-performance liquid chromatography tandem mass spectrometry in EDTA plasma collected from 2,275 participants (age 60 ± 8 years, mean ± SD; 49% women, 605 (27%) with type 2 diabetes mellitus) of the Maastricht Study, an observational, population-based cohort study. Cross-sectional associations were assessed using multivariable linear regression analysis adjusting for age, sex, mean arterial pressure (MAP), heart rate, lifestyle factors, and medication. Since arterial stiffness measures are intrinsically pressure dependent, we additionally assessed the associations with pressure-corrected counterparts, instead of statistically correcting for MAP.

**RESULTS:**

Fasting dicarbonyl concentrations were associated with arterial stiffness measures (greater cfPWV, cPWV, and cYEM) in most crude models, but not in adjusted models. The use of pressure-corrected metrics did not materially change the association of interest.

**CONCLUSIONS:**

Fasting plasma concentrations of either MGO, GO, or 3-DG are not independently associated with arterial stiffness in this cross-sectional analysis.

## Graphical Abstract

**Graphical Abstract:**
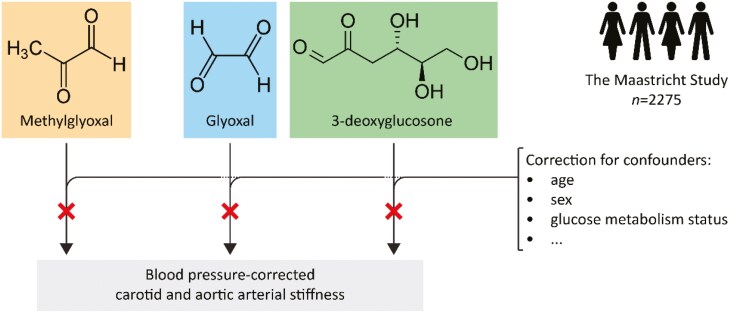

Large artery stiffening is an independent predictor of cardiovascular disease (CVD) and cardiovascular mortality in the general population.^1–3^ Although arterial stiffening is an age-related process, it appears to be accelerated in patients with type 2 diabetes mellitus (T2DM), leading to an increase in vascular complications and a considerably higher risk of cardiovascular mortality.^4^ Although the exact mechanisms underlying the accelerated arterial stiffening is incompletely understood, the potential role of advanced glycation end products (AGEs) has emerged.

AGEs are stable irreversible adducts that are formed by a non-enzymatic reaction of reduced sugars with proteins.^5^ Several clinical studies have shown an association between AGEs and arterial stiffness,^6,7^ with a more pronounced association under hyperglycemic conditions such as in T2DM.^7^ AGEs may contribute to arterial stiffening by (increased) cross-linking of collagen in the extracellular matrix (ECM), which alters the vascular ultrastructure, and by triggering inflammatory and oxidative pathways which contribute to endothelial dysfunction and vascular smooth muscle cell activation.^8,9^ Although high blood glucose levels have been assigned as a key mediator in AGEs formation, there is now considerable evidence that dicarbonyls are the most important precursors in the formation of AGEs.^8^ Dicarbonyls, such as methylglyoxal (MGO), glyoxal (GO), and 3-deoxyglucosone (3-DG) are highly reactive intermediates derived from glucose and lipid oxidation. These dicarbonyls are increased in patients with diabetes mellitus and hypertension.^8^ Dicarbonyls mainly react with lysine and arginine residues in proteins, which leads to the formation of AGEs. Dicarbonyls in vascular tissue might trigger maladaptive responses in vascular tissue, changing biomechanical properties of the arterial wall, resulting in decreased flexibility of the artery.

Previous studies in small animals^6,10–12^ have demonstrated a role for dicarbonyls as contributors to arterial stiffness. However, the association between plasma dicarbonyl levels and arterial stiffening has not been studied on a large scale in human.^13^ The aim of the present study is to investigate the cross-sectional association between fasting plasma dicarbonyl levels and arterial stiffening in participants from The Maastricht Study. Since arterial stiffness indices are intrinsically pressure dependent, we additionally performed a participant-specific blood pressure correction to make a reliable statement on pressure-independent effects.^14,15^

## Methods

### The Maastricht Study

We used data from The Maastricht Study, an observational prospective population-based cohort study. The rationale and methodology of The Maastricht Study have been described elsewhere.^16^ In brief, the study focuses on the cause, pathophysiology, complications, and comorbidities of T2DM and is characterized by an extensive phenotyping approach. Eligible for participation were all individuals aged between 40 and 75 years and living in the southern part of the Netherlands. Participants were recruited through mass media campaigns and from the municipal registries and the regional Diabetes Patient Registry via mailings. Recruitment was stratified according to known T2DM status, with an oversampling of individuals with T2DM, for reasons of statistical efficiency.

The present report includes cross-sectional data from the first 3,451 participants, who completed the baseline survey between November 2010 and September 2013. The examinations of each participant were performed within a time window of 3 months. The study has been approved by the institutional medical ethical committee (NL31329.068.10) and the Minister of Health, Welfare, and Sports of the Netherlands (Permit 131088-105234-PG). All participants gave written informed consent.

### Quantification of fasting dicarbonyl levels

Participants underwent blood collection after overnight fasting. Quantification of MGO, GO, and 3-DG in EDTA plasma was performed via ultra-performance liquid chromatography tandem mass spectrometry (UPLC-MS/MS) as described previously.^17^ Plasma samples were stored at −80 °C until needed for analysis. For the analysis, EDTA plasma samples (25 μL) were mixed with 75 μL of d_8_-O-phenylenediamine (oPD; 10 mg of oPD in 10 mL of 1.6 mol/L perchloric acid). After an overnight reaction at room temperature while shielded from light, 10 μL of internal standard stock solution was added. Samples were mixed and subsequently centrifuged for 20 min at 21,000 *g* at a temperature of 4 °C; 10 μL was injected for UPLC-MS/MS analysis. Inter-assay variations for MGO, GO, and 3-DG were 4.3%, 5.1% and 2.2%, respectively.^18^

### Arterial stiffness measurements

All macrovascular measurements were performed by a trained technician unaware of the participant’s clinical status, in a dark, quiet, temperature-controlled room (21–23 °C). All measurements were performed with the participant in a supine position after 10 min of rest. Participants were asked to refrain from smoking and from drinking coffee, tea, or alcoholic beverages 3 h prior to the measurements. Brachial systolic, diastolic, and mean arterial pressure (MAP) were recorded every 5 min with an oscillometric device (Accutorr Plus, Datascope Inc., Montvale, NJ, USA). The mean MAP and mean heart rate (HR) of these measurements were used in the statistical analysis. A three-lead electrocardiogram was recorded continuously during the measurements to facilitate automatic signal processing.

#### Carotid-to-femoral pulse wave velocity.

Carotid-to-femoral pulse wave velocity (cfPWV) was determined according to recent guidelines^19^ with the use of applanation tonometry (SphygmoCor, Atcor Medical, Sydney, Australia). Pressure waveforms were determined at the right common carotid and right common femoral arteries. Difference in the time of pulse arrival from the R-wave of the electrocardiogram between the two sites (transit time) was determined with the intersecting tangents algorithm. The pulse wave travel distance was calculated as 80% of the direct straight distance (measured with an infantometer) between the two arterial sites. The median of three consecutive cfPWV (defined as travel distance/transit time) recordings was used in the analyses. A pressure-corrected index based on cfPWV was calculated as previously described,^15^ however using the measured cfPWV instead of a heart-ankle pulse wave velocity.^20^

#### Local carotid elastic properties.

Indices of carotid stiffness were measured at the left common carotid artery (10 mm proximal to the carotid bulb), with the use of an ultrasound scanner equipped with a 7.5-MHz linear probe (MyLab 70, Esaote Europe B.V., Maastricht, the Netherlands). This set-up enables the measurement of diameter, distension, and intima-media thickness (IMT) as described previously.^21,22^ Briefly, during the ultrasound measurements, a B-mode image based on 19 M-lines was depicted on screen. An online echo-tracking algorithm showed real-time anterior and posterior wall displacements. The M-mode recordings were composed of 19 simultaneous recordings at a frame rate of 498 Hz. The distance between the M-line recording positions was 0.96 mm; thus, a total segment of 18.24 mm of each artery was covered by the scan plane. For offline processing, the radiofrequency signal, sampled at 50 MHz, was fed into a dedicated computer-based acquisition system (ART.LAB, Esaote Europe B.V. Maastricht, the Netherlands). Data processing was performed in MATLAB R2007b (MathWorks, Natick, Massachusetts, USA). We obtained the distension waveforms from the radiofrequency data with the use of a wall track algorithm.^21^ We defined carotid IMT as the distance in the posterior wall from the leading-edge interface between lumen and intima to the leading-edge interface between media and adventitia during diastole.^22^ Diameter was defined as the distance between the trailing edge of the anterior and the leading edge of the posterior wall. Media-adventitia echoes were obtained during diastole. Distension was defined as the difference between systolic and diastolic diameter. We used the median diameter, distension, and IMT of three consecutive measurements in the analyses.

Local arterial elastic properties were quantified through the calculation of the following indices^23^:

Local carotid pulse wave velocity (cPWV), estimated using the Bramwell–Hill equation^24^:


$$\begin{array}{l} cPWV=\sqrt{\frac{P_{s}-P_{d}}{D_{s}-D_{d}}\cdot \frac{D_{d}}{2\rho }}, \end{array}$$ (1)

with *P*_s_ and *P*_d_ the systolic and diastolic blood pressure; *D*_s_ and *D*_d_ the systolic and diastolic carotid diameter; and *ρ* the blood mass density, taken to be 1,050 kg/m^3^.^25^

Carotid Young’s elastic modulus (cYEM)^26^:


$$\begin{array}{l} cYEM=\frac{cPWV^{2}\cdot D_{d}\cdot \rho }{IMT}, \end{array}$$ (2)

Pressure-corrected measures of local carotid artery elastic properties were calculated assuming an exponential pressure–diameter relationship, as previously described.^14,15^

### Covariates

Measurements of general covariates within the Maastricht are extensively described elsewhere.^16^ Weight, height, body mass index (BMI), and waist circumference were measured during physical examination. History of CVD, physical activity, alcohol consumption, and smoking status (never, former, current) were assessed using a questionnaire. Use of lipid modifying, glucose-lowering, and/or antihypertensive medication was checked during a medication interview. MAP, and mean HR were obtained during vascular measurements. In addition, ambulatory 24-h MAP and HR were measured. The lipid profile was determined from fasting venous blood sampling. Glucose metabolism status (GMS) was categorized into normal glucose metabolism (NGM), impaired glucose metabolism (IGM), and T2DM according to the World Health Organization 2006 criteria. The estimated glomerular filtration rate was computed with the CKD-EPI (Chronic Kidney Disease Epidemiology Collaboration) formula, using serum creatinine and cystatin C.^16^ The Dutch Healthy Diet index was calculated from dietary intake data obtained via a questionnaire.

### Statistical Analysis

Comparisons of general characteristics per plasma dicarbonyl level (in tertiles) were made by ANOVA or X^2^-tests. Variables with a skewed distribution were log_10_ transformed before the analysis. Arterial stiffness was compared between participants with different GMS. These results are reported as unstandardized βs with corresponding 95% confidence intervals (CIs). For the main analysis we conducted multivariable linear regression to study the associations between fasting plasma MGO (nmol/L), 3-DG (nmol/L), and GO (nmol/L) and arterial stiffness (cfPWV (m/s), cYEM (MPa), and cPWV (m/s), and their pressure-corrected counterparts). We report standardized βs with corresponding 95% CIs. Adjustments were done for GMS and for a series of cardiovascular risk factors to evaluate their influence on the association of the dicarbonyls with cfPWV and local carotid artery stiffness.

Model 1 was a crude model, which included only MGO, 3-DG, or GO as a determinant; model 2 was additionally adjusted for age, sex, and GMS. Because of the dependency of carotid stifffness measurements on blood pressure, model 3 was additionally adjusted for MAP and mean HR during vascular measurements, and use of antihypertensive drugs. The associations in model 3 were repeated with 24-h ambulatory blood pressure and mean HR measurements instead of the ones obtained during vascular measurements. Model 4 was additionally adjusted for known confounders of stiffness i.e., BMI, smoking status, physical activity, use of lipid-modifying medication, fasting triglycerides and total-to-high-density lipoprotein cholesterol levels, alcohol use, history of CVD, estimated glomerular filtration rate (eGFR) and Dutch Healthy Diet (DHD) score.

Furthermore, antihypertensive medication was split into renin-angiotensin-system (RAS)-inhibitors and other types of antihypertensive medication. Finally, the analysis was repeated with waist circumference instead of BMI in model 4. We tested the potential interaction between plasma dicarbonyl levels and sex,^27^ age,^28^ and GMS—because of an oversampling of participants with T2DM in our cohort. *P*-values < 0.05 were considered statistically significant.

We performed a full-case analysis. All analyses were performed with the Statistical Package for Social Sciences (version 26.0; IBM, Chicago, Illinois, USA).

## Results

### Study Population

**Figure 1.**
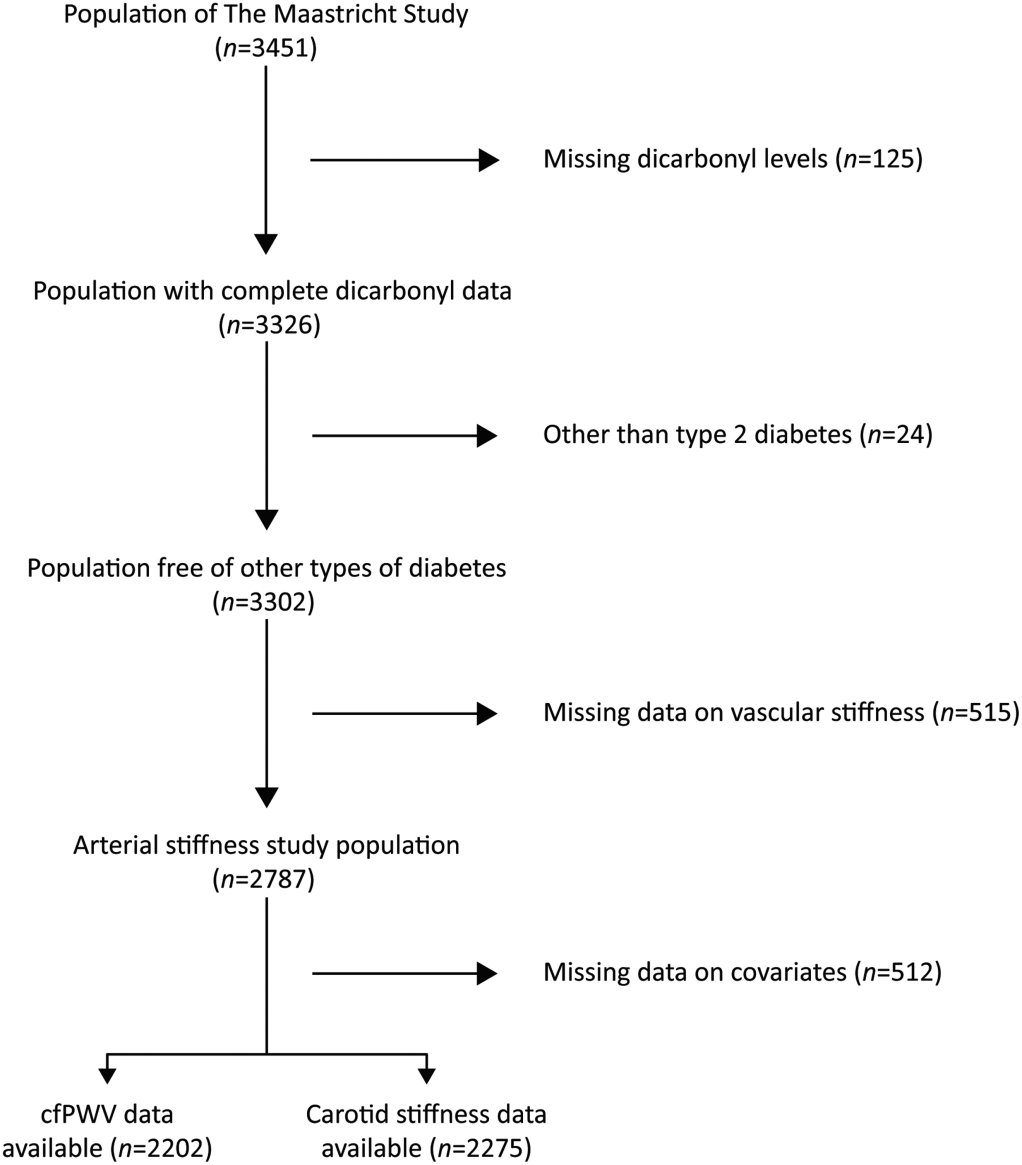
Flow diagram delineating the selection of the study population. Abbreviation: cfPWV, carotid-to-femoral pulse wave velocity.

The total study population comprised 3,451 participants, from which 125 were excluded because of missing fasting dicarbonyl levels. Subsequently, 24 participants were excluded because of having other types of diabetes than T2DM. From the remaining, 515 participants were excluded because vascular stiffness measure(s) were missing. Since we performed a full-case analysis, all participants with missing confounders were removed from the analysis (*n* = 512). Finally, since outcome and covariate data were not available in all individuals the number of individuals included in the different regression analyses varied (*n* = 2,202 for cfPWV, *n* = 2,275 for local stiffness measures; **Figure 1**).

**Tables 1–3** show the general characteristics of participants in the final study population, stratified according to their MGO, GO, or 3-DG tertiles. In general, patients in the highest tertiles of the dicarbonyls were older, and were predominantly male in the MGO and 3-DG group, but predominantly female in the GO group. They had a less favorable cardiometabolic risk profile indicated by a higher BMI, MAP, mean HR and serum triglyceride levels, and a lower eGFR. They received antihypertensive treatment more often compared to participants in the lowest tertiles. In addition, more participants in the highest tertiles suffered from T2DM and were known with CVD. Furthermore, they were less physical active, consumed more alcohol and had a less healthy diet.

**Table 1.** Clinical characteristics of the study population according to tertiles of plasma methylglyoxal levels

Characteristics	Total population (*n* = 2,275)	Plasma methylglyoxal tertiles			*P*-value
		Lowest (*n* = 763)	Middle (*n* = 754)	Highest (*n* = 758)	
**Demographics**					
Age (years)	60 ± 8.0	59 ± 8	60 ± 8	61 ± 8	<0.001
Men	1,160 (51%)	375 (49%)	389 (52%)	396 (52%)	0.445
**Lifestyle**					
Smoking (%)					0.027
Never	790 (35%)	283 (37%)	259 (34%)	248 (33%)	
Former	1,217 (53%)	411 (54%)	390 (52%)	416 (55%)	
Current	268 (12%)	69 (9%)	105 (14%)	94 (12%)	
Body mass index (kg/m^2^)	27.1 ± 4.5	26.6 ± 4.3	26.9 ± 4.4	27.6 ± 4.7	<0.001
Physical activity (h/week)	13.0 [8.3;18.5]	13.0 [8.3;18.3]	13.5 [8.5;18.8]	12.5 [7.8;18.8]	0.01
Dutch Healthy Diet index	83.5 ± 14.6	84.5 ± 14.6	83.4 ± 14.4	82.6 ± 14.6	0.04
**Biological**					
Mean arterial pressure	97 ± 10	95 ± 10	97 ± 10	98 ± 10	<0.001
Heart rate	63 ± 9	62 ± 9	62 ± 9	64 ± 10	<0.001
Total-to-HDL cholesterol ratio	3.5 [2.8;4.3]	3.4 [2.7;4.3]	3.5 [2.9;4.3]	3.6 [2.9;4.4]	0.01
Triglycerides, mmol/L	1.2 [0.9;1.7]	1.1 [0.8;1.6]	1.2 [0.9;1.7]	1.3 [1.0;1.9]	<0.001
eGFR, ml/min/1.73 m^2^	88.0 ± 14.7	91.4 ± 12.7	87.6 ± 14.0	85.0 ± 16.3	<0.001
Diabetes status					<0.001
No diabetes	1,324 (58%)	532 (70%)	451 (60%)	341 (45%)	
Pre-diabetes	346 (15%)	112 (15%)	111 (15%)	123 (16%)	
Type 2 diabetes	605 (27%)	119 (16%)	192 (25%)	294 (39%)	
History of CVD	373 (16%)	112 (15%)	115 (15%)	146 (19%)	0.032
**Medication**					
Lipid-modifying medication use	816 (36%)	213 (28%)	272 (36%)	331 (44%)	<0.001
Antihypertensive medication use (% yes)	878 (39%)	211 (28%)	304 (40%)	363 (48%)	<0.001
Betablocker	413 (18%)	104 (14%)	130 (17%)	179 (24%)	<0.001
diuretic	367 (16%)	82 (11%)	111 (15%)	174 (23%)	<0.001
calcium antagonist	223 (10%)	46 (6%)	79 (10%)	98 (13%)	<0.001
Renin-angiotensin system inhibitors	652 (29%)	158 (21%)	223 (30%)	271 (36%)	<0.001
Angiotensin-converting-enzyme inhibitors	259 (11%)	60 (8%)	90 (12%)	109 (14%)	<0.001
Angiotensin-II inhibitors	395 (17%)	98 (13%)	134 (18%)	163 (22%)	<0.001
Renin inhibitors	4 (0.2%)	1 (0.1%)	0 (0%)	3 (0.4%)	0.173
**Outcome measures**					
Methylglyoxal (nmol/L)	322 [279;377]	265 [244;279]	323 [305;337]	404 [377;456]	<0.001
Glyoxal (nmol/L)	1,208 [1018;1469]	1,093 [946;1289]	1,222 [1042;1434]	1,341 [1095;1726]	<0.001
3-DG (nmol/L)	1,256 [1100;1495]	1,160 [1051;1319]	1,252 [1102;1488]	1,407 [1202;1732]	<0.001
cfPWV (m/s)	8.6 [7.5;10.0]	8.3 [7.4;9.5]	8.6 [7.5;10.1]	9.0 [7.7;10.4]	<0.001
Corrected cfPWV (m/s)	8.8 [7.8;10.2]	8.5 [7.7;9.7]	8.8 [7.8;10.3]	9.2 [7.9;10.5]	<0.001
cPWV (m/s)	8.6 ± 1.7	8.4 ± 1.6	8.6 ± 1.7	8.9 ± 1.8	<0.001
Corrected cPWV (m/s)	8.6 ± 1.5	8.5 ± 1.5	8.5 ± 1.5	8.8 ± 1.6	<0.001
Carotid YEM (MPa)	0.67 [0.53;0.88]	0.65 [0.51;0.83]	0.66 [0.51;0.87]	0.72 [0.56;0.96]	<0.001
Corrected carotid YEM (MPa)	0.68 [0.55;0.87]	0.67 [0.53;0.85]	0.67 [0.53;0.85]	0.71 [0.58;0.92]	<0.001

Data presented as mean ± standard deviation, median [interquartile range], or *n* (%). Abbreviations: HDL, high-density-lipoprotein; eGFR, estimated glomerular filtration rate; CVD, cardiovascular disease; cfPWV, carotid-to-femoral pulse wave velocity; YEM, Young’s elastic modulus; cPWV, carotid pulse wave velocity.

**Table 2.** Clinical characteristics of the study population according to tertiles of plasma 3-deoxyglucosone (3-DG) levels

Characteristics	Total population (*n* = 2275)	Plasma 3-deoxyglucosone tertiles			*P*-value
		Lowest (*n* = 762)	Middle (*n* = 763)	Highest (*n* = 750)	
**Demographics**					
Age (years)	60.0 ± 8.0	58 ± 8	60 ± 8	62 ± 8	<0.001
Men	1160 (51%)	276 (36%)	378 (50%)	506 (67%)	<0.001
**Lifestyle**					
Smoking (%)					<0.001
Never	790 (35%)	486 (64%)	385 (50%)	244 (33%)	
Former	1217 (53%)	303 (40%)	264 (35%)	223 (30%)	
Current	268 (12%)	378 (50%)	415 (54%)	424 (57%)	
Body mass index (kg/m^2^)	27.1 ± 4.5	81 (11%)	84 (11%)	103 (14%)	<0.001
Physical activity (h/week)	13.0 [8.3;18.5]	25.3 ± 3.6	26.7 ± 4.2	29.1 ± 4.7	<0.001
Dutch Healthy Diet index	83.5 ± 14.6	8.4 [2.2;17.3]	9.1 [2.0;19.8]	7.5 [0.7;18.9]	<0.001
**Biological**					
Mean arterial pressure	97 ± 10	95 ± 11	96 ± 10	99 ± 10	<0.001
Heart rate	63 ± 9	61 ± 8	62 ± 9	65 ± 11	<0.001
Total-to-HDL cholesterol ratio	3.5 [2.8;4.3]	3.2 [2.6;4.0]	3.5 [2.9;4.3]	3.7 [3.0;4.5]	<0.001
Triglycerides, mmol/L	1.2 [0.9;1.7]	1.1 [0.8;1.4]	1.2 [0.9;1.6]	1.4 [1.1;2.1]	<0.001
eGFR, ml/min/1.73 m^2^	88.0 ± 14.7	89.8 ± 12.8	88.2 ± 14.3	86.0 ± 16.5	<0.001
Diabetes status					<0.001
- No diabetes	1,324 (58%)	675 (89%)	530 (69%)	119 (16%)	
- Pre-diabetes	346 (15%)	73 (10%)	141 (18%)	132 (18%)	
- Type 2 diabetes	605 (27%)	14 (2%)	92 (12%)	499 (67%)	
History of CVD	373 (16%)	82 (11%)	129 (17%)	162 (22%)	<0.001
**Medication**					
Lipid-modifying medication use	816 (36%)	146 (19%)	213 (28%)	457 (61%)	<0.001
Antihypertensive medication use (% yes)	878 (39%)	183 (24%)	243 (32%)	452 (60%)	<0.001
Betablocker	413 (18%)	79 (10%)	122 (16%)	212 (28%)	<0.001
Diuretic	367 (16%)	64 (8%)	96 (13%)	207 (28%)	<0.001
Calcium antagonist	223 (10%)	32 (4%)	56 (7%)	135 (18%)	<0.001
Renin-angiotensin system inhibitors	652 (29%)	119 (16%)	176 (23%)	357 (48%)	<0.001
Angiotensin-converting-enzyme inhibitors	259 (11%)	37 (5%)	74 (10%)	148 (20%)	<0.001
Angiotensin-II inhibitors	395 (17%)	81 (11%)	103 (13%)	211 (28%)	<0.001
Renin inhibitors	4 (0.2%)	1 (0.1%)	0 (0%)	3 (0.4%)	0.167
**Outcome measures**					
Methylglyoxal (nmol/L)	322 [279;377]	296 [263;339]	316 [276;367]	356 [308;416]	<0.001
Glyoxal (nmol/L)	1,208 [1018;1469]	1,258 [1063;1532]	1,161 [982;1400]	1,200 [1014;1469]	<0.001
3-DG (nmol/L)	1,256 [1100;1495]	1,051 [994;1101]	1,258 [1199;1325]	1,640 [1498;1922]	<0.001
cfPWV (m/s)	8.6 [7.5;10.0]	8.1 [7.3;9.3]	8.5 [7.4;9.8]	9.3 [8.0;10.8]	<0.001
Corrected cfPWV (m/s)	8.8 [7.8;10.2]	8.4 [7.6;9.5]	8.8 [7.7;10.0]	9.4 [8.2;10.9]	<0.001
cPWV (m/s)	8.6 ± 1.7	8.3 ± 1.6	8.5 ± 1.6	9.0 ± 1.8	<0.001
Corrected cPWV (m/s)	8.6 ± 1.5	8.4 ± 1.5	8.5 ± 1.5	8.9 ± 1.6	<0.001
Carotid YEM (MPa)	0.67 [0.53;0.88]	0.63 [0.49;0.81]	0.67 [0.52;0.87]	0.75 [0.57;0.98]	<0.001
Corrected carotid YEM (MPa)	0.68 [0.55;0.87]	0.65 [0.52;0.83]	0.68 [0.55;0.86]	0.73 [0.58;0.95]	<0.001

Data presented as mean ± standard deviation, median [interquartile range], or *n* (%). Abbreviations: HDL, high-density-lipoprotein; eGFR, estimated glomerular filtration rate; CVD, cardiovascular disease; cfPWV, carotid-to-femoral pulse wave velocity; YEM, Young’s elastic modulus; cPWV, carotid pulse wave velocity.

**Table 3.** Clinical characteristics of the study population according to tertiles of plasma glyoxal levels

Characteristics	Total population (*n* = 2,275)	Plasma glyoxal tertiles			*P*-value
		Lowest (*n* = 764)	Middle (*n* = 760)	Highest (*n* = 751)	
**Demographics**					
Age (years)	60.0 ± 8.0	59 ± 8	60 ± 8	61 ± 8	<0.001
Sex (men %)	1,160 (51%)	423 (55%)	391 (51%)	346 (46%)	0.001
**Lifestyle**					
Smoking (%)					0.267
Never	790 (35%)	260 (34%)	275 (36%)	255 (34%)	
Former	1,217 (53%)	401 (52%)	397 (52%)	419 (56%)	
Current	268 (12%)	103 (13%)	88 (12%)	77 (10%)	
Body mass index (kg/m^2^)	27.1 ± 4.5	27.6 ± 4.5	27.0 ± 4.3	26.5 ± 4.5	<0.001
Physical activity (h/week)	13.0 [8.3;18.5]	13.0 [7.8;18.5]	13.0 [8.4;18.5]	13.0 [8.3;19.0]	0.640
Dutch Healthy Diet index	83.5 ± 14.6	82.1 ± 15.0	83.6 ± 13.9	84.8 ± 14.6	0.001
**Biological**					
Mean arterial pressure	97 ± 10	96 ± 10	96 ± 10	98 ± 11	0.023
Heart rate	63 ± 9	63 ± 10	63 ± 10	63 ± 9	0.989
Total-to-HDL cholesterol ratio	3.5 [2.8;4.3]	3.6 [3.0;4.4]	3.5 [2.9;4.4]	3.3 [2.7;4.1]	<0.001
Triglycerides, mmol/L	1.2 [0.9;1.7]	1.2 [0.9;1.7]	1.2 [0.9;1.7]	1.2 [0.9;1.7]	0.203
eGFR, mL/min/1.73 m^2^	88.0 ± 14.7	88.9 ± 14.6	88.1 ± 14.0	87.1 ± 15.3	0.054
Diabetes status					0.347
- No diabetes	1324 (58%)	434 (57%)	449 (59%)	441 (59%)	
- Pre-diabetes	346 (15%)	124 (16%)	122 (16%)	100 (13%)	
- Type 2 diabetes	605 (27%)	206 (27%)	189 (25%)	210 (28%)	
History of CVD (yes%)	373 (16%)	134 (17.7%)	112 (14.8%)	127 (16.7%)	0.350
**Medication**					
Lipid-modifying medication use	816 (36%)	294 (38%)	258 (34%)	264 (35%)	0.161
Antihypertensive medication use (% yes)	878 (39%)	301 (39%)	286 (38%)	291 (39%)	0.774
Betablocker	413 (18%)	145 (19%)	132 (17%)	136 (18%)	0.717
diuretic	367 (16%)	122 (16%)	123 (16%)	122 (16%)	0.988
calcium antagonist	223 (10%)	77 (10%)	70 (9%)	76 (10%)	0.797
Renin-angiotensin system inhibitors	652 (29%)	227 (30%)	215 (28%)	210 (28%)	0.725
Angiotensin-converting-enzyme inhibitors	259 (11%)	90 (12%)	87 (11%)	82 (11%)	0.868
Angiotensin-II inhibitors	395 (17%)	138 (18%)	128 (17%)	129 (17%)	0.810
Renin inhibitors	4 (0.2%)	0 (0%)	2 (0.3%)	2 (0.3%)	0.363
**Outcome measures**					
Methylglyoxal (nmol/L)	322 [279;377]	295 [262;347]	317 [279;364]	352 [302;413]	<0.001
Glyoxal (nmol/L)	1,208 [1018;1469]	943 [831;1019]	1,210 [1138;1283]	1,640 [1471;1927]	<0.001
3-DG (nmol/L)	1,256 [1100;1495]	1,291 [1136;14912]	1,239 [1096;1473]	1,226.1 [1077;1524]	0.496
cfPWV (m/s)	8.6 [7.5;10.0]	8.6 [7.6;9.8]	8.6 [7.4;10.0]	8.6 [7.5;10.1]	0.808
Corrected cfPWV (m/s)	8.8 [7.8;10.2]	8.8 [7.8;10.0]	8.9 [7.8;10.2]	8.8 [7.7;10.2]	0.947
cPWV (m/s)	8.6 ± 1.7	8.5 ± 1.7	8.6 ± 1.6	8.7 ± 1.8	0.079
Corrected cPWV (m/s)	8.6 ± 1.5	8.5 ± 1.6	8.6 ± 1.4	8.7 ± 1.6	0.186
Carotid YEM (MPa)	0.67 [0.53;0.88]	0.67 [0.51;0.87]	0.67 [0.53;0.87]	0.69 [0.53;0.89]	0.172
Corrected carotid YEM (MPa)	0.68 [0.55;0.87]	0.67 [0.53;0.87]	0.69 [0.55;0.86]	0.69 [0.56;0.88]	0.303

Data presented as mean ± standard deviation, median [interquartile range], or *n* (%). Abbreviations: HDL, high-density-lipoprotein; eGFR, estimated glomerular filtration rate; CVD, cardiovascular disease; cfPWV, carotid-to-femoral pulse wave velocity; YEM, Young’s elastic modulus; cPWV, carotid pulse wave velocity.

### Glucose Metabolism Status and Stiffness Measures

Participants with T2DM had stiffer arteries compared to participants with NGM. This was predominantly seen for cfPWV. cfPWV was significantly higher in participants with T2DM compared to participants with NGM (9.5 [8.3;11.1] vs. 8.2 [7.3;9.3] m/s, *P* < 0.001) and IGM (8.8 [7.8;10.1] m/s, *P* < 0.001). cfPWV did not significantly differ between NGM and IGM (*P* = 0.243).

This was also the case after individual blood pressure correction (**Supplementary Table S1** online) Local carotid elastic properties did not differ between participants with different GMS in the fully adjusted model (**Supplementary Table S1**).

### Fasting Dicarbonyls Levels and Arterial Stiffness Measures

**Figure 2.**
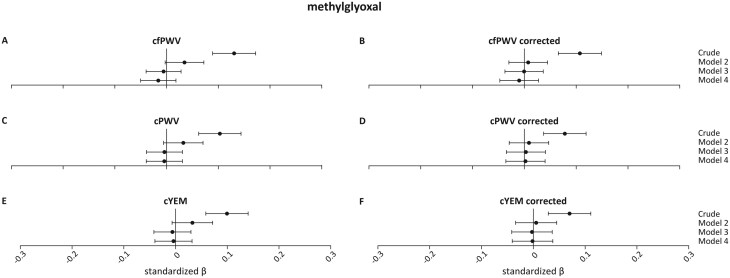
Association between methylglyoxal and stiffness measurements, and their pressure-corrected counterparts. Regression results are presented as standardized *β*s (95% CIs). Model 1 is the crude model; model 2 = crude model + age, sex, and glucose metabolism status; model 3 = model 2 + mean arterial pressure (MAP) and mean heart rate (HR) during vascular measurements, and use of antihypertensive drugs; model 4 = model 3 + body mass index, smoking status, physical activity, use of lipid-modifying medication, fasting triglycerides and total-to-high-density lipoprotein cholesterol levels, alcohol use, history of CVD, estimated glomerular filtration rate and Dutch Healthy Diet index. Abbreviations: cfPWV, carotid-to-femoral pulse wave velocity; cPWV, carotid pulse wave velocity; cYEM, carotid Young’s elastic modulus.

**Figure 2** shows the associations of MGO with cfPWV, Cpwv, and cYEM and their pressure-corrected counterparts. Fasting MGO levels were statistically significantly associated with cfPWV, cPWV, and cYEM in the crude models, but these associations did not remain statistically significant after adjusting for age, sex, and GMS (cfPWV *β*: 0.035 (−0.003;0.072), *P* = 0.07; cPWV *β*: 0.032 (−0.006;0.071), *P* = 0.097; cYEM *β*: 0.033 (−0.007;0.072), *P* = 0.104). Participant-specific blood pressure correction did not lead to different results (**Figure 2**).

**Figure 3.**
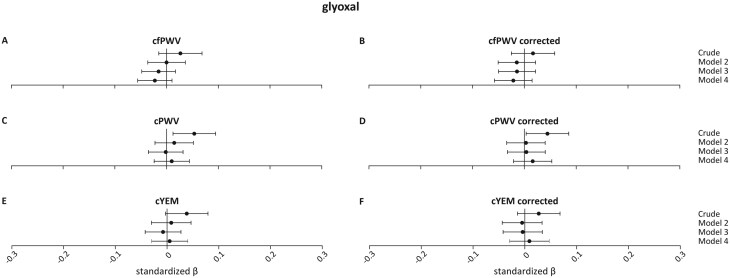
Association between glyoxal and carotid stiffness measurements and their pressure-corrected counterparts. Regression results are presented as standardized *β*s (95% CIs). Model 1 is the crude model; model 2 = crude model + age, sex, and glucose metabolism status; model 3 = model 2 + mean arterial pressure (MAP) and mean heart rate (HR) during vascular measurements, and use of anti-hypertensive drugs; model 4 = model 3 + body mass index, smoking status, physical activity, use of lipid-modifying medication, fasting triglycerides and total-to-high-density lipoprotein cholesterol levels, alcohol use, history of CVD, estimated glomerular filtration rate and Dutch Healthy Diet index. Abbreviations: cfPWV, carotid-to-femoral pulse wave velocity; cPWV, carotid pulse wave velocity; cYEM, carotid Young’s elastic modulus.

**Figure 3** shows the association of GO with cfPWV, cPWV, and cYEM and their pressure-corrected counterparts. There was a statistically significant associations between fasting GO level and cPWV in the crude model (cPWV *β*: 0.054 (0.012;0.095), *P* = 0.011) but this association did not remain statistically significant after adjusting for age, sex, and GMS (cPWV *β*: 0.015 (−0.022;0.052), *P* = 0.436). There were no statistically significant associations between plasma fasting GO levels and cfPWV, or cYEM in the crude models (cfPWV *β*: 0.027 (−0.015;0.069), *P* = 0.211; cYEM *β*: 0.038 (−0.003;0.079), *P* = 0.069). Participant-specific blood pressure correction did not lead to different results (**Figure 3**).

**Figure 4.**
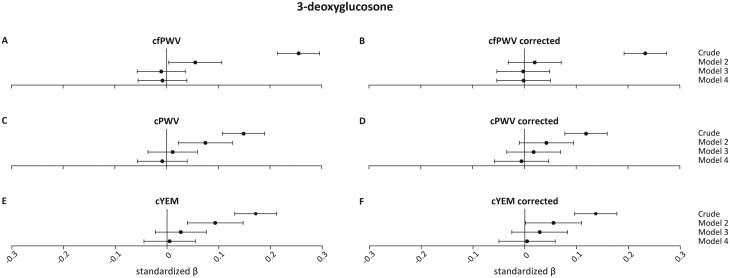
Association between 3-deoxyglucosone (3-DG) and carotid stiffness measurements and their pressure corrected counterparts. Regression results are presented as standardized *β*s (95% CIs). Model 1 is the crude model; model 2 = crude model + age, sex, and glucose metabolism status; model 3 = model 2 + mean arterial pressure (MAP) and mean heart rate (HR) during vascular measurements, and use of anti-hypertensive drugs; model 4 = model 3 + body mass index, smoking status, physical activity, use of lipid-modifying medication, fasting triglycerides and total-to-high-density lipoprotein cholesterol levels, alcohol use, history of CVD, estimated glomerular filtration rate and Dutch Healthy Diet index. Abbreviations: cfPWV, carotid-to-femoral pulse wave velocity; cPWV, carotid pulse wave velocity; cYEM, carotid Young’s elastic modulus.

**Figure 4** shows a statistically significant association between fasting 3-DG levels and cfPWV, Cpwv, and cYEM in the crude models (cfPWV *β*: 0.255 (0.215;0.296), *P* < 0.001; cPWV *β*: 0.149 (0.108;0.190), *P* < 0.001; cYEM *β*: 0.171 (0.131;0.212), *P* < 0.001). These associations did not remain statistically significant after adjusting for age, sex, GMS, MAP, and mean HR during vascular measurements, and use of antihypertensive drugs. Participant-specific blood pressure correction did not lead to different results (**Figure 4**).

For all dicarbonyls, the crude associations were substantially attenuated after adjustments in model 2. However, there is a notable difference between the corrected and uncorrected measurements. In the crude model, the standardized *β* of the corrected measures is larger than the standardized *β* of the uncorrected measures. This suggests that the effect of blood pressure is “hidden” in the uncorrected measures. This effect seems to disappear in model 3 where the *β*’s are similar again. We repeated the regression analysis to solely compare the methods of blood pressure corrections. To achieve this, we split model 3 in the uncorrected measures in model 3a; model 2 + mean MAP, and model 3b; model 3a + mean HR and antihypertensive medication. We compared model 3a of the uncorrected measures to model 2 of the corrected measures. Standardized *β*’s were approximately the same (data not shown).

### Interaction Analyses

There was no statistically significant interaction between fasting plasma dicarbonyl levels and sex. In the minimally adjusted model, it seemed that GMS affected the associations of GO and 3-DG with cfPWV and cPWV, both corrected and uncorrected, but this was attenuated in the fully adjusted model (*P* > 0.05, data not shown). There was a significant interaction between age and both GO and 3-DG with cfPWV. A stratified analysis based on the median age of the participants (61 years), did not reveal a stronger effect within one of the groups (**Supplementary Table S2** online).

### Additional Analysis

Adjustments for 24 h MAP and 24 h HR measurements instead of MAP and HR during vascular measurements did not materially affect the results (**Supplementary Table S3.1**), nor did using waist circumference instead of BMI (**Supplementary Table S3.2**). In addition, specifying antihypertensive medication into RAS-inhibitors and other types of antihypertensive medication did not change the results (**Supplementary Table S3.3**).

## Discussion

In this cross-sectional study we found that fasting plasma concentrations of either MGO, GO, or 3-DG are not independently associated with indices of arterial stiffness. Participant-specific blood pressure correction did not materially change these results.

We recently showed, in a smaller subset of the current study population, that plasma pentosidine levels and accumulation of AGEs in the skin, as measured via skin autofluorescence, are associated with aortic stiffening. This result was more pronounced in patients with T2DM.^7^ The present study extends our previous research by studying the associations of precursors in the formation of AGE and arterial stiffness. When it comes to dicarbonyls, most clinical studies focus on plasma MGO levels in patients with diabetes, in which fasting levels of MGO are 2- to 3-fold higher compared to healthy individuals.^12^ Previous work highlighted the importance of mainly MGO-derived AGEs in the development of vascular complications in individuals with diabetes.^8,9^ Two prospective cohort studies found an association between plasma MGO and incident CVD in patients with type 1 and type 2 diabetes,^29,30^ although such associations of MGO with prior CVD and atherosclerosis were not found in a recent cross-sectional analysis.^31^ The lack of associations with prior cardiovascular events may be due to limitations of cross-sectional analyses, such as inclusion of case subjects only with favorable outcome at baseline and susceptible to interventions to treat CVD. An earlier study also found only prospective associations, but no cross-sectional association, between plasma MGO levels and macroangiopathy, measured as cIMT, cfPWV, and systolic blood pressure.^13^ Nevertheless, this indicates that cross-sectional analyses should be interpreted with caution. Thus, based on our cross-sectional analysis and its shortcomings, we cannot exclude the possibility that dicarbonyls are associated with the development of arterial stiffness in prospective cohort studies.

It has recently been shown that incremental glucose peaks are associated with aortic stiffness and maladaptive carotid remodeling, but not with carotid stiffness.^32^ Furthermore, it has been suggested that postprandial dicarbonyl levels, rather than fasting levels, are more strongly associated with macrovascular damage.^33^ Therefore, it remains to be elucidated whether postprandial dicarbonyl peaks are more harmful/strongly associated compared to chronic elevated plasma levels when it comes to vascular stiffness, although a recent report from the Maastricht Study did not find consistent interactions between fasting and post-OGTT plasma dicarbonyl levels and prior CVD.

When comparing arterial stiffness measures between individuals with normal glucose metabolism, impaired glucose metabolism and diabetes, we observed that only cfPWV and pressure-corrected cfPWV, differed between groups (**Supplementary Table S1** online). This might indicate that arterial stiffening is a heterogeneous process, affecting the relatively elastic aorta earlier than the more muscular carotid artery. Stiffness of the aorta, as assessed by magnetic resonance imaging, has been reported as a stronger predictor of cfPWV, peripheral SBP, and pulse pressure than carotid stiffness in healthy individuals.^34^

### Blood Pressure Correction

In this study, we also evaluated the merit of individual blood pressure correction compared to population-based blood pressure correction. Statistical correction may simultaneously correct for (i) the acute dependence of arterial stiffness of blood pressure at the time of the measurement, and (ii) the remodeling effect of blood pressure on arterial stiffness.^35^ Correction based on individual arterial properties and blood pressures, as recorded during measurements, may help to better distinguish both aspects, while it only considers effect 1 by definition. Hence, in this study we have presented both approaches alongside one another. For the uncorrected carotid stiffness measures, we statistically corrected for blood pressure effects by adjusting for MAP in model 3. For the corrected carotid stiffness measures, the correction does not depend on the association within the study population but on individual properties.

We showed that the effect of blood pressure is “hidden” in the uncorrected measures, as the standardized *β* of the corrected measures is larger than the standardized *β* of the uncorrected measures. Indeed, when splitting model 3 in the uncorrected measures in model 3a; model 2 + mean MAP, and model 3b; model 3a + mean HR and antihypertensive medication, we observed that the standardized *β*’s of model 3a were similar to the standardized *β* of model 2 for uncorrected measures. Both approaches appear equivalent in this particular study, although the similarity in the model behavior is far from proof that both approaches are equivalent.

As shown in **Figures 2–4** the effect is attenuated when going from the crude model to model 2. We performed a separate analysis including confounders in the regression analysis one-by-one. This revealed age as the major contributor to arterial stiffness outcomes. This might explain why we do not see a large effect of our blood pressure correction in model 3.

Our results support that pressure corrections are highly relevant. In clinical practice, patient-specific blood pressure correction could be beneficial especially for follow-up measurements. However, further research is needed to substantiate the merit of the corrected measures.

### Perspectives

Fasting plasma dicarbonyl levels were not independently associated with arterial stiffness measures in this cross-sectional analysis. The use of participant-specific blood pressure correction did not alter this conclusion. Future research should aim to understand the complex and heterogeneous effects of dicarbonyls on the arterial wall. Direct measurements of dicarbonyls in the arterial wall and follow-up studies are of major importance to elucidate pathophysiological and clinical implications of dicarbonyls in arterial stiffness.

## Supplementary Data

Supplementary materials are available at *American Journal of Hypertension* (http://ajh.oxfordjournals.org).

hpaf068_suppl_Supplementary_Tables_S1-S3

## Data Availability

Data are available from The Maastricht Study for any researcher who meets the criteria for access to confidential data. The corresponding author may be contacted to request data.

## Non-standard Abbreviations and Acronyms:

3-DG: 3-deoxyglucosone
AGE: advanced glycation end product
BMI: body mass index
cE: carotid Young’s elastic modulus
cPWV: carotid pulse wave velocity
cfPWV: carotid-to-femoral pulse wave velocity
CVD: cardiovascular disease
DHD-score: Dutch Healthy Diet score
eGFR: estimated glomerular filtration rate
GO: glyoxal
GMS: glucose metabolism status
IGM: impaired glucose metabolism
IMT: intima-media thickness
MAP: mean arterial pressure
MGO: methylglyoxal
NGM: normal glucose metabolism
OGTT: oral glucose tolerance test
RAS-inhibitor: renin-angiotensin-system inhibitor
T2DM: type 2 diabetes mellitus
UPLC-MS: ultra-performance liquid chromatography tandem mass spectrometry
