# Supplementary material for: Plasma Reactive Dicarbonyls Are Not Independently Associated With Arterial Stiffness: The Maastricht Study
Source: Am J Hypertens. 2025 May 16;38(10):777–87. doi: 10.1093/ajh/hpaf068 (PMC12448583; doi:10.1093/ajh/hpaf068)
Supplement: hpaf068_suppl_Supplementary_Tables_S1-S3 [file hpaf068_suppl_supplementary_tables_s1-s3.doc]

**Plasma reactive dicarbonyls are not independently associated with arterial stiffness: The Maastricht Study**

**Supplementary digital content 1**

Supplementary tables:

**Table S1. Association between glucose metabolism status and arterial stiffness measures.**

|  | NGM | **IGM** | **T2DM** | IGM | **T2DM** |
| --- | --- | --- | --- | --- | --- |
| cfPWV (m/s) | reference | 0.01 (0.00 ; 0.01) | **0.03 (0.02 ; 0.04)*** | reference | **0.02 (0.01 ; 0.03)*** |
| cPWV (m/s) | reference | -0.06 (-0.23 ; 0.10) | 0.07 (-0.10 ; 0.24) | reference | 0.13 (-0.06 ; 0.32) |
| cYEM (MPa) | reference | -0.01 (-0.03 ; 0.01) | 0.00 (-0.02 ; 0.01) | reference | 0.01 (-0.01 ; 0.03) |
|  |  |  |  |  |  |
| cfPWV corrected (m/s) | reference | 0.01 (0.00 ; 0.02) | **0.04 (0.03 ; 0.05)*** | reference | **0.03 (0.01 ; 0.04)*** |
| cPWV corrected (m/s) | reference | -0.04 (-0.21 ; 0.12) | 0.07 (-0.10 ; 0.23) | reference | 0.11 (-0.08 ; 0.30) |
| cYEM corrected (MPa) | reference | -0.01 (-0.03 ; 0.01) | 0.00 (-0.02 ; 0.02) | reference | 0.01 (-0.01 ; 0.03) |

Regression results are presented as unstandardized βs (95% CIs). Models are fully adjusted for glucose metabolism status, age, sex, mean arterial pressure (for uncorrected arterial stiffness measures only), mean heart rate, use of anti-hypertensive drugs, body mass index, smoking status, physical activity, use of lipid modifying medication, fasting triglycerides and total-to-high-density lipoprotein cholesterol levels, alcohol use, history of cardiovascular disease, estimated glomerular filtration rate and Dutch-healthy diet index. *n*=2275 for carotid stiffness measures*, n*=2202 for aortic stiffness measures. NGM, normal glucose metabolism; IGM, impaired glucose metabolism; T2DM, type 2 diabetes mellitus; cfPWV, carotid-to-femoral pulse wave velocity; cPWV, carotid pulse wave velocity; cYEM, carotid Young’s elastic modulus. **P*<0.05 was considered statistically significant.

**Table S2. Regression analysis stratified by age.**

|  |  | **cfPWV**  **(age < 61 years)** | | **cfPWV**  **(age > 61 years)** | |
| --- | --- | --- | --- | --- | --- |
|  |  | Standardized β | *p*-value | Standardized β | *p*-value |
| Glyoxal | model 1 | -0.009 (-0.058;0.040) | 0.722 | 0.012 (-0.048;0.072) | 0.699 |
|  | model 2 | -0.015 (-0.062;0.033) | 0.541 | 0.029 (-0.029;0.086) | 0.329 |
|  | model 3 | -0.021 (-0.064;0.021) | 0.321 | 0.000 (-0.052;0.051) | 0.985 |
|  | model 4 | -0.039 (-0.081;0.004) | 0.078 | -0.010 (-0.063;0.042) | 0.699 |
|  |  |  |  |  |  |
| 3-deoxyglucosone | model 1 | **0.200 (0.151;0.249)** | **<0.001** | 0,213 (0,157;0,269) | **<0.001** |
|  | model 2 | 0.062 (-0.005;0.129) | 0.068 | 0,018 (-0,062;0,097) | 0.664 |
|  | model 3 | 0.000 (-0.061;0.061) | 0.998 | -0,008 (-0,086;0,071) | 0.850 |
|  | model 4 | 0.006 (-0.056;0.067) | 0.861 | -0,006 (-0,086;0,073) | 0.879 |

Regression results are presented as standardized coefficient βs (95% Cis). Model 1 is the crude model; model 2=crude model + sex, and glucose metabolism status; model 3=model 2 + mean arterial pressure and mean heart rate during vascular measurements, and use of anti-hypertensive drugs; model 4=model 3 + waist circumference, smoking status, physical activity, use of lipid modifying medication, fasting triglycerides and total-to-high-density lipoprotein cholesterol levels, alcohol use, history of cardiovascular disease, estimated glomerular filtration rate and Dutch-healthy diet index. cfPWV, carotid-to-femoral pulse wave velocity. *P*<0.05 was considered statistically significant.

**Table S3.1** Sensitivity analysis waist circumference instead of BMI.

|  |  | **cfPWV** | | **cPWV** | | **cYEM** | |
| --- | --- | --- | --- | --- | --- | --- | --- |
|  |  | Standardized β | *p*-value | Standardized β | *p*-value | Standardized β | *p*-value |
| Methylglyoxal | model 1 | **0.130 (0.089;0.172)** | **<0.001** | **0.103 (0.062;0.144)** | **<0.001** | **0.099 (0.058;0.140)** | **<0.001** |
|  | model 2 | 0.035 (-0.003;0.072) | 0.070 | 0.032 (-0.006;0.071) | 0.097 | 0.033 (-0.007;0.072) | 0.104 |
|  | model 3 | -0.006 (-0.040;0.028) | 0.735 | -0.004 (-0.039;0.030) | 0.812 | -0.006 (-0.042;0.030) | 0.739 |
|  | model 4 | -0.016 (-0.050;0.019) | 0.371 | -0.006 (-0.041;0.029) | 0.748 | -0.005 (-0.041;0.031) | 0.774 |
|  |  |  |  |  |  |  |  |
| Glyoxal | model 1 | 0.027 (-0.015;0.069) | 0.209 | **0.054 (0.013;0.095)** | **0.011** | 0.038 (-0.003;0.079) | 0.069 |
|  | model 2 | 0.000 (-0.036;0.036) | 0.997 | 0.015 (-0.022;0.052) | 0.435 | 0.008 (-0.030;0.046) | 0.677 |
|  | model 3 | -0.015 (-0.048;0.017) | 0.354 | -0.002 (-0.035;0.032) | 0.926 | -0.008 (-0.042;0.027) | 0.662 |
|  | model 4 | -0.021 (-0.054;0.012) | 0.215 | 0.006 (-0.028;0.040) | 0.716 | 0.001 (-0.034;0.036) | 0.942 |
|  |  |  |  |  |  |  |  |
| 3-deoxyglucosone | model 1 | **0.255 (0.215;0.296)** | **<0.001** | **0.149 (0.108;0.190)** | **<0.001** | **0.171 (0.131;0.212)** | **<0.001** |
|  | model 2 | **0.056 (0.004;0.107)** | **0.034** | **0.075 (0.023;0.128)** | **0.005** | **0.093 (0.039;0.147)** | **0.001** |
|  | model 3 | -0.010 (-0.057;0.036) | 0.671 | 0.012 (-0.036;0.060) | 0.623 | 0.027 (-0.023;0.076) | 0.289 |
|  | model 4 | -0.010 (-0.057;0.037) | 0.679 | -0.004 (-0.052;0.045) | 0.877 | 0.008 (-0.042;0.058) | 0.741 |

Association between methylglyoxal, glyoxal, and 3-deoxyglucosone, and carotid stiffness measurements. Regression results are presented as standardized βs (95% CIs). Model 1 is the crude model; model 2=crude model + age, sex, and glucose metabolism status; model 3=model 2 + mean arterial pressure and mean heart rate during vascular measurements, and use of anti-hypertensive drugs; model 4=model 3 + waist circumference, smoking status, physical activity, use of lipid modifying medication, fasting triglycerides and total-to-high-density lipoprotein cholesterol levels, alcohol use, history of cardiovascular disease, estimated glomerular filtration rate and Dutch-healthy diet index. cfPWV, carotid-to-femoral pulse wave velocity; cPWV, pulse wave velocity; cYEM, Young’s elastic modulus. *P*<0.05 was considered statistically significant.

**Table S3.2** Sensitivity analysis 24hour blood pressure and heart rate measurements instead of office measurements.

|  |  | **cfPWV** | | **cPWV** | | **cYEM** | |
| --- | --- | --- | --- | --- | --- | --- | --- |
|  |  | Standardized β | *p*-value | Standardized β | *p*-value | Standardized β | *p*-value |
| Methylglyoxal | model 1 | **0.129 (0.085;0.172)** | **<0.001** | **0.098 (0.054;0.141)** | **<0.001** | **0.089 (0.046;0.133)** | **<0.001** |
|  | model 2 | 0.033 (-0.006;0.073) | 0.099 | 0.025 (-0.016;0.066) | 0.230 | 0.021 (-0.021;0.063) | 0.322 |
|  | model 3 | 0.012 (-0.026;0.050) | 0.549 | 0.007 (-0.032;0.046) | 0.721 | 0.002 (-0.039;0.042) | 0.929 |
|  | model 4 | 0.004 (-0.035;0.042) | 0.846 | 0.010 (-0.029;0.049) | 0.624 | 0.007 (-0.033;0.047) | 0.729 |
|  |  |  |  |  |  |  |  |
| Glyoxal | model 1 | 0.030 (-0.014;0.074) | 0.187 | **0.054 (0.011;0.098)** | **0.015** | 0.036 (-0.008;0.079) | 0.107 |
|  | model 2 | 0.005 (-0.033;0.043) | 0.798 | 0.013 (-0.026;0.053) | 0.509 | 0.005 (-0.036;0.046) | 0.814 |
|  | model 3 | 0.006 (-0.031;0.042) | 0.754 | 0.013 (-0.025;0.051) | 0.498 | 0.005 (-0.034;0.044) | 0.794 |
|  | model 4 | -0.001 (-0.038;0.037) | 0.970 | 0.027 (-0.012;0.065) | 0.171 | 0.020 (-0.019;0.059) | 0.321 |
|  |  |  |  |  |  |  |  |
| 3-deoxyglucosone | model 1 | **0.253 (0.210;0.296)** | **<0.001** | **0.133 (0.089;0.176)** | **<0.001** | **0.160 (0.116;0.203)** | **<0.001** |
|  | model 2 | **0.058 (0.003;0.113)** | **0.038** | **0.059 (0.003;0.115)** | **0.039** | **0.082 (0.024;0.140)** | **0.005** |
|  | model 3 | 0.017 (-0.035;0.070) | 0.516 | 0.021 (-0.033;0.075) | 0.436 | 0.043 (-0.013;0.099) | 0.133 |
|  | model 4 | 0.018 (-0.035;0.071) | 0.502 | 0.001 (-0.054;0.055) | 0.984 | 0.020 (-0.036;0.076) | 0.478 |

Association between methylglyoxal, glyoxal, and 3-deoxyglucosone, and carotid stiffness measurements. Regression results are presented as standardized βs (95% CIs). Model 1 is the crude model; model 2=crude model + age, sex, and glucose metabolism status; model 3=model 2 + mean arterial pressure and mean heart rate from 24 hour measurements, and use of anti-hypertensive drugs; model 4=model 3 + body mass index, smoking status, physical activity, use of lipid modifying medication, fasting triglycerides and total-to-high-density lipoprotein cholesterol levels, alcohol use, history of cardiovascular disease, estimated glomerular filtration rate and Dutch-healthy diet index. cfPWV, carotid-to-femoral pulse wave velocity; cPWV, pulse wave velocity; cYEM, Young’s elastic modulus. *P*<0.05 was considered statistically significant.

**Table S3.3** Sensitivity analysis renin-angiotensin system inhibitors instead of all types of anti-hypertensive medication.

|  |  | **cfPWV** | | **cPWV** | | **cYEM** | |
| --- | --- | --- | --- | --- | --- | --- | --- |
|  |  | Standardized β | *p*-value | Standardized β | *p*-value | Standardized β | *p*-value |
| Methylglyoxal | model 1 | **0.131 (0.089;0.172)** | **<0.001** | **0.103 (0.062;0.144)** | **<0.001** | **0.099 (0.059;0.140)** | **<0.001** |
|  | model 2 | 0.035 (-0.003;0.072) | 0.070 | 0.032 (-0.006;0.071) | 0.097 | 0.033 (-0.007;0.072) | 0.104 |
|  | model 3 | -0.004 (-0.038;0.029) | 0.795 | -0.004 (-0.039;0.030) | 0.806 | -0.005 (-0.041;0.031) | 0.783 |
|  | model 4 | -0.015 (-0.049;0.019) | 0.378 | -0.005 (-0.040;0.030) | 0.776 | -0.004 (-0.040;0.032) | 0.831 |
|  |  |  |  |  |  |  |  |
| Glyoxal | model 1 | 0.027 (-0.015;0.069) | 0.211 | **0.054 (0.012;0.095)** | **0.011** | 0.038 (-0.003;0.079) | 0.069 |
|  | model 2 | 0.000 (-0.036;0.036) | 0.996 | 0.015 (-0.022;0.052) | 0.436 | 0.008 (-0.030;0.046) | 0.677 |
|  | model 3 | -0.015 (-0.048;0.018) | 0.367 | -0.002 (-0.035;0.032) | 0.926 | -0.007 (-0.042;0.027) | 0.676 |
|  | model 4 | -0.022 (-0.056;0.011) | 0.183 | 0.010 (-0.024;0.044) | 0.576 | 0.005 (-0.030;0.040) | 0.779 |
|  |  |  |  |  |  |  |  |
| 3-deoxyglucosone | model 1 | **0.255 (0.215;0.296)** | **<0.001** | **0.149 (0.108;0.190)** | **<0.001** | **0.171 (0.131;0.212)** | **<0.001** |
|  | model 2 | **0.055 (0.004;0.107)** | **0.034** | **0.075 (0.023;0.127)** | **0.005** | **0.093 (0.039;0.147)** | **0.001** |
|  | model 3 | -0.010 (-0.057;0.036) | 0.669 | 0.012 (-0.036;0.059) | 0.634 | 0.027 (-0.023;0.076) | 0.290 |
|  | model 4 | -0.008 (-0.055;0.039) | 0.739 | -0.008 (-0.057;0.040) | 0.740 | 0.005 (-0.045;0.055) | 0.843 |

Association between methylglyoxal, glyoxal, and 3-deoxyglucosone, and carotid stiffness measurements. Regression results are presented as standardized βs (95% CIs). Model 1 is the crude model; model 2=crude model + age, sex, and glucose metabolism status; model 3=model 2 + mean arterial pressure and mean heart rate during vascular measurements, and use of renin-angiotensin system inhibitors; model 4=model 3 + body mass index, smoking status, physical activity, use of lipid modifying medication, fasting triglycerides and total-to-high-density lipoprotein cholesterol levels, alcohol use, history of cardiovascular disease, estimated glomerular filtration rate and Dutch-healthy diet index. cfPWV, carotid-to-femoral pulse wave velocity; cPWV, pulse wave velocity; cYEM, Young’s elastic modulus. *P*<0.05 was considered statistically significant.
